# Optimization Methods in Information and Management Sciences

**DOI:** 10.1155/2015/498951

**Published:** 2015-03-10

**Authors:** Jung-Fa Tsai, C. Alec Chang, Chih-Chou Chiu, Steve Hsueh-Ming Wang

**Affiliations:** ^1^Department of Business Management, National Taipei University of Technology, No. 1, Section 3, Chung-Hsiao East Road, Taipei 10608, Taiwan; ^2^Department of Industrial & Manufacturing Systems Engineering, University of Missouri, Columbia, MO 65211, USA; ^3^Engineering, Science, and Project Management Department, University of Alaska Anchorage, 3211 Providence Drive, Anchorage, AK 99508-4614, USA

This special issue continues to raise the aim of The Scientific World Journal in the operations research subject area through its cutting-edge presentation of advanced research, carefully developed applications, and innovative technology components. This special issue also provides a forum for researchers and practitioners to review and disseminate quality research work on optimization methods and their applications in the context of information and management sciences and to identify critical issues for further developments. This special issue involves original papers, selected by the editors and related to the various researches themes on optimization methods in information and management sciences. These papers are related to various subjects such as computer science and electric engineering, computational intelligence, big data, security, management strategy, data envelopment analysis, facility location problem, production planning, and workflow nets. The research area represented by the papers accepted for the special issue should be of interest for a wide scientific community because of its multidisciplinary character. The accepted papers of this special issue are briefly described as below.

J. W.-S. Hu et al. provide several notable findings from the empirical studies. First, the expected temperature shock significantly and positively affects both the near-month and far-month natural gas and heating oil futures returns. This suggests that the investors should invest a portion of their funds on the weather derivative commodities for their hedging and arbitraging purposes. Next, significant temperature shock effect on both the conditional mean and volatility of natural gas and heating oil prices suggests that when the market expects the degree days in the future to be higher than average, energy demand increases, increasing the near-month and far-month natural gas and heating oil futures returns. The nonlinear temperature effects exist for natural gas futures returns, suggesting the market expects the degree day volatility to increase with natural gas demand. Also, the results indicate that expected inventory surprises significantly and negatively affect the far-month natural gas futures returns only. Moreover, volatility of natural gas futures returns is higher on Thursdays and that of near-month heating oil futures returns is higher on Wednesdays than other days, coinciding with the release of the weekly natural gas and heating oil storage report by the Energy Information Administration (EIA), respectively. Additionally, storage announcement for natural gas significantly affects near-month and far-month natural gas futures returns. However, storage effect does not exist for heating oil futures returns. Furthermore, both natural gas and heating oil futures returns are affected more by the weighted average temperature reported by multiple weather reporting stations than that reported by a single weather reporting station.

“Applying Different Independent Component Analysis Algorithms and Support Vector Regression for IT Chain Store Sales Forecasting” by W. Dai et al. utilizes three different ICA methods including spatial ICA (sICA), temporal ICA (tICA), and spatiotemporal ICA (stICA) to extract features from the sales data and compare their performance in sales forecasting of IT chain store. Experimental results from real sales data show that the sales forecasting scheme by integrating stICA and SVR outperforms the comparison models in terms of forecasting error. The stICA is a promising tool for extracting effective features from branch sales data and the extracted features can improve the prediction performance of SVR for sales forecasting.

“Assessment and Classification of Service Learning: A Case Study of CS/EE Students” by H.-Y. Kao et al. investigates the undergraduate students in computer science/electric engineering (CS/EE) in Taiwan to measure their perceived benefits from the experiences in service learning coursework. In addition, the confidence in their professional disciplines and its correlation with service learning experiences are examined. The results show that students take positive attitudes toward service learning and their perceived benefits from service learning are correlated with their confidence in professional disciplines. Furthermore, this study designs the knowledge model by Bayesian network (BN) classifiers and term frequency-inverse document frequency (TFIDF) for counseling students on the optimal choice of service learning.

Y.-C. Hu uses the multilayer perceptron to construct a robust nonlinear interval regression model using the genetic algorithm (GA) without estimating the vexed degree of contamination. Those suspected outliers determined by GA beyond or beneath the data interval simply impose slight effect on the determination of upper and lower bounds for a cost function. Simulation results demonstrate that the proposed method performs well for contaminated datasets.

The paper “Analyzing Big Data with the Hybrid Interval Regression Methods” by C.-H. Huang et al. collaborates interval regression with the smooth support vector machine (SSVM) to analyze big data. Recently, the smooth support vector machine (SSVM) was proposed as an alternative to the standard SVM that has been proved more efficient than the traditional SVM in processing large-scale data. In addition, the soft margin method is proposed to modify the excursion of separation margin and to be effective in the gray zone.

“A Secure Operational Model for Mobile Payments” by T.-K. Chang presents the design of a secure operational model for mobile payments in which access control is based on a service-oriented architecture. A customer uses his/her mobile device to get authorization from a remote server and generate a two-dimensional barcode as the payment certificate. This payment certificate has a time limit and can be used once only. The system also provides the ability to remotely lock and disable the mobile payment service.

The paper “Advantage Management Strategy in Competition via Technological Race Perspective: Empirical Evidence from the Taiwanese Manufacturing Industry” by T.-Y. Hung et al. investigates the advantage management strategies of a firm regarding the technological race in the manufacturing sector. This is to reveal whether firms adopt a catch-up or leapfrogging strategy in the competition for innovation. The results show that competition is fierce in the Taiwanese manufacturing industry. Taiwanese manufacturing firms (mostly SMEs) tend to adopt the “catch-up” strategy to keep up with their competitors in order to remain in the technological race. The result indicates that, under financial constraints, Taiwanese manufacturing firms attempt to invest in R&D to catch up with their rivals or to avoid being eliminated from the race.

“A Study of the Strategic Alliance for EMS Industry: The Application of a Hybrid DEA and GM (1, 1) Approach” by C. N. Wang et al. aims to develop effective methods to assist enterprise to measure the firms' operation efficiency, find out the candidate priority under several different inputs and outputs, and forecast the values of those variables in the future. The methodologies are constructed by the concepts of data envelopment analysis (DEA) and grey model (GM). Realistic data in four consecutive years (2009–2012) of total of 20 companies of the electronic manufacturing service (EMS) industry that went public are completely collected. This paper tries to help target company, DMU1, to find the right alliance partners. By our proposed approach, the results show the priority in the recent years. The research study is hopefully of interest to managers who are in manufacturing industry in general and EMS enterprises in particular.

The paper “A Simulated Annealing Methodology to Multiproduct Capacitated Facility Location with Stochastic Demand” by J. Qin et al. proposes a nonlinear mixed integer programming model whose objective is to minimize the total cost, including transportation cost, inventory cost, operation cost, and setup cost. A combined simulated annealing (CSA) algorithm is presented to solve the model, in which the outer layer subalgorithm optimizes the facility location decision and the inner layer subalgorithm optimizes the demand allocation based on the determined facility location decision. The results obtained with this approach have shown that the CSA is a robust and practical approach for solving a multiple product problem, which generates the suboptimal facility location decision and inventory policies. Meanwhile, we also found that the transportation cost and the demand deviation are the stronger influencing factors on the optimal decision than others.

“A Two-Stage Method to Determine Optimal Product Sampling considering Dynamic Potential Market” by Z. Hu et al. develops an optimization model for the diffusion effects of free samples under dynamic changes in potential market based on the characteristics of independent product and presents a two-stage method to figure out the sampling level. The impact analysis of the key factors on the sampling level shows that the increase of the external coefficient or internal coefficient has a negative influence on the sampling level. And the changing rate of the potential market has no significant influence on the sampling level whereas the repeat purchase has a positive one. Using logistic analysis and regression analysis, the global sensitivity analysis gives a whole analysis of the interaction of all parameters, which provides a two-stage method to estimate the impact of the relevant parameters in the case of inaccuracy of the parameters and to be able to construct a 95% confidence interval for the predicted sampling level. Finally, the paper provides the operational steps to improve the accuracy of the parameter estimation and an innovational way to estimate the sampling level.

The paper “The Equivalency between Logic Petri Workflow Nets and Workflow Nets” by J. Wang et al. proposes logic Petri workflow nets (LPWNs) based on logic Petri nets (LPNs) which can describe and analyze batch processing functions and passing value indeterminacy in cooperative systems. Process mining is regarded as an important bridge between modeling and analysis of data mining and business process. Workflow nets (WF-nets) are the extension to Petri nets (PNs) and have successfully been used to process mining. Some shortcomings cannot be avoided in process mining, such as duplicate tasks, invisible tasks, and the noise of logs. The online shop in electronic commerce in this paper is modeled to prove the equivalence between LPWNs and WF-nets, and advantages of LPWNs are presented.

“Understanding Transferable Supply Chain Lessons and Practices to a “High-Tech” Industry Using Guidelines from a Primary Sector Industry: A Case Study in the Food Industry Supply Chain” by A. E. C. Mondragon et al. identifies lessons and practices that comprise innovations in the supply chain of a firm in a perceived “low-tech” industry that can be used to provide guidelines in the design of the supply chain of a “high-tech” industry, in this case composite materials. This work uses the case study/site visit with analogy methodology to collect data from a Spanish leading producer of fresh fruit juice which is sold in major European markets and makes use of a cold chain. The study highlights supply base management and visibility/traceability as two elements of the supply chain in a “low-tech” industry that can provide guidelines that can be used in the configuration of the supply chain of the composite materials industry.

K.-Y. Chen et al. propose a novel virtual goods recommendation method based on these social interactions. The contact strength and contact influence result from interactions with social neighbors and influence users' buying intention. Their research highlights the importance of social interactions in virtual goods recommendation. The experiment's data were retrieved from an online virtual worlds platform, and the results show that the proposed method, considering social interactions and social life circle, has better performance than existing recommendation methods.

C. N. Wang et al. integrate DEA models and grey system theory to evaluate Indian electricity industry past-to-future performance. Their study expects the rankings of the current Indian electricity companies under control of the Ministry of Power. In the meantime, this research would like to test whether any significant differences exist between two DEA models: Malmquist nonradial and radial. Then, one advance model of MPI would be chosen to see these companies' performance in recent years and some next few years by using forecasting results of grey system theory. Totally, 14 items of realistic data are considered to be involved in this evaluation after the strict selection from the whole industry. Finally, the results found that all companies in the industry have not shown many abrupt changes on their scores and it is always not consistently good or consistently standing out, which demonstrated the high applicable usability of the integrated methods.

“An Extended EPQ-Based Problem with a Discontinuous Delivery Policy, Scrap Rate, and Random Breakdown” by S. W. Chiu et al. extends recent work using an economic production quantity- (EPQ-) based inventory model with a continuous inventory issuing policy, defective items, and machine breakdown by incorporating a multiple delivery policy into the model to replace the continuous policy and investigates the effect on the optimal run time decision for this specific EPQ model. Then they further expand the scope of the problem to combine the retailer's stock holding cost into our study. This enhanced EPQ-based model can be used to reflect the situation found in contemporary manufacturing firms in which finished products are delivered to the producer's own retail stores and stocked there for sale. A second model is developed and studied. With the help of mathematical modeling and optimization techniques, the optimal run times that minimize the expected total system costs comprising costs incurred in production units, transportation, and retail stores are derived for both models. Numerical examples are provided to demonstrate the applicability of the research results.

